# The frequencies of peripheral blood CD5^+^CD19^+^ B cells, CD3^−^CD16^+^CD56^+^ NK, and CD3^+^CD56^+^ NKT cells and serum interleukin-10 in patients with multiple sclerosis and neuromyelitis optica spectrum disorder

**DOI:** 10.1186/s13223-021-00596-5

**Published:** 2022-01-14

**Authors:** Leila Khani, Mir Hadi Jazayeri, Reza Nedaeinia, Mahmood Bozorgmehr, Seyed Masood Nabavi, Gordon A. Ferns

**Affiliations:** 1 Department of Immunology, School of Medicine, Iran University of Medical Science, Shahid Hemmat Highway, P.O Box 14665-354, 14496-14535 Tehran, Iran; 2 Immunology Research Center, Iran University of Medical Science, Shahid Hemmat Highway, P.O Box 14665-354, 14496-14535 Tehran, Iran; 3 Pediatric Inherited Diseases Research Center, Research Institute for Primordial Prevention of Non-Communicable Disease, Isfahan University of Medical Sciences, Isfahan, Iran; 4 Oncopathology Research Center, Iran University of Medical Science, Tehran, Iran; 5 Department of Regenerative Biomedicine, Cell Science Research Center, Neuroscience and Cognition Center, Royan Institute for Stem Cell Biology and Technology, ACECR, Tehran, Iran; 6 Division of Medical Education, Brighton and Sussex Medical School, Falmer, Brighton, BN1 9PH Sussex UK

**Keywords:** B cells, IL-10, MS, NMOSD, NK cells

## Abstract

**Background:**

Multiple sclerosis (MS) and neuromyelitis optica syndrome disease (NMOSD) are inflammatory diseases of the central nervous system. The pathogenesis and treatments for these two conditions are very different. Natural killer (NK) and natural killer T (NKT) cells are immune cells with an important role in shaping the immune response. B cells are involved in antigen presentation as well as antibody and cytokine production. There is conflicting evidence of the roles of NK, NKT, and B cells in the two conditions. We aimed to compare the frequency of CD3^−^CD16^+^CD56^+^NK, CD3^+^ CD56^+^ NKT, and CD5^+^CD19^+^ B cells in the peripheral blood and serum Interleukin-10 (IL-10) in patients with MS and NMOSD.

**Methods:**

CD19^+^CD5^+^ B, CD3^−^ CD16^+^CD56^+^ NK, and CD3^+^CD56^+^ NKT cells were quantitated by flow cytometry in 15 individuals with Interferon-Beta (IFN-β) treated relapsing–remitting MS (RRMS), 15 untreated RRMS, and 15 NMOSD patients as well as 30 healthy controls (HC). Serum IL-10 was measured using an enzyme-linked immunosorbent assay (ELISA).

**Results:**

The percentage of CD3^−^CD56^+^CD16^+^ NK cells in the peripheral blood of IFN-treated MS (1.81 ± 0.87) was significantly lower than for untreated RRMS (4.74 ± 1.80), NMOSD (4.64 ± 1.26) and HC (5.83 ± 2.19) (p < 0.0001). There were also differences for the percentage of CD3^−^CD16^+^ and CD3^−^CD56^+^ cells (p < 0.001 and p < 0.0007; respectively). IFN-treated RRMS (2.89 ± 1.51) had the lowest proportion of CD3^+^CD56^+^ among the study groups (p < 0.002). Untreated RRMS (5.56 ± 3.04) and NMOSD (5.47 ± 1.24) had higher levels of CD3^+^CD56^+^ than the HC (3.16 ± 1.98). The mean percentage of CD19^+^CD5^+^ B cells in the peripheral blood of untreated RRMS patients (1.32 ± 0.67) was higher compared to the patients with NMOSD (0.30 ± 0.20), HC (0.5 ± 0.22) and IFN-treated RRMS (0.81 ± 0.17) (p < 0.0001). Serum interleukin-10 was significantly higher in the IFN-treated RRMS (8.06 ± 5.39) and in HC (8.38 ± 2.84) compared to untreated RRMS (5.07 ± 1.44) and the patients with NMOSD (5.33 ± 2.56) (p < 0.003).

**Conclusions:**

The lower proportion of CD3^−^CD56^+^ CD16^+^ NK and CD3^+^CD56^+^ cells in peripheral blood of IFN-treated RRMS compared to other groups suggests the importance of immunomodulation in patients with RRMS disorder. Based on the differences in CD19^+^CD5^+^ B cells and serum IL-10 between patients and HC, supplementary assessments could be of value in clarifying their roles in autoimmunity.

## Introduction

MS is a common neuro-inflammatory disease affecting young people globally. Of the four diagnostic types of MS, RRMS is the most prevalent accounting for 85% of MS cases. Primary progressive, secondary progressive and primary relapsing MS are other types of this disorder [1]. Specific genetic and environmental factors increase the susceptibility of individuals to MS [1]. NMOSD is another neuro-inflammatory disease [2], It is associated with similar clinical signs as MS, which include vision problems and motor disabilities. Studies have shown glycosylated lesions, nerve damage and atrophy of the spinal cord as the clinical symptoms in patients with NMOSD. As the clinical manifestations of NMOSD disease are comparable to those of MS, NMOSD has been misdiagnosed as a type of MS for many years until the identification of an anti NMO antibody known as anti-aquaporin (AQP4) in 2004 [3, 4]. NK cells play a key role in immune surveillance and defense against viral infections. NK cells possess no specific receptor and recognize their targets without sensitization, and exert their effects via cytokine release and direct cytotoxicity mediated by granzyme A and perforin [5]. There is conflicting evidence concerning the roles of NK cells in the pathogenesis and disease protection in the experimental autoimmune encephalomyelitis (EAE) mouse model. NK cells shape immune responses and induce the polarization of different subsets of the central nervous system (CNS) infiltrating dendritic cells. However, many studies on EAE have suggested that the role of the NK cells is mediated through changes in the population of executable cells that prevent tissue damage [6, 7]. This controversy is related to the function of NK cells in the onset and progression of the disease. The two following points may explain this paradox. Firstly, NK cells play a substantial role in T helper (Th) polarization and consequently in the onset and progression of MS [8]. Secondly, different subtypes of NK cells probably have different functions in the onset and progression of EAE. Studies on RRMS patients have shown that the frequency of these cells is reduced in MS [9] and elevates following immunotherapy. These results point to the remarkable role of these immune cells in RRMS [10, 11]. However, the effects of NK cells on the immunological responses in MS have not yet been elucidated. There is evidence suggesting that NK cells may have a pathogenic role in some CNS disease. NK cells migrate to the CNS and affect immune system components. CD3^+^CD56^+^ NKT cells are innate immune cells that express different antigens including: CD3, CD56 and some chemokine receptors. They are located in the liver, bone marrow, thymus, spleen, and peripheral blood [12]. NKT cell recognize lipid antigen via MHC-I like molecule CD1d and activate dendritic cells following by innate and adoptive immune response stimulation or regulation [13]. Activated NKT cell secret different cytokines including IFN-γ, IL-2, -4, -10, -13, -17, -21, and 22, granulocyte–macrophage colony-stimulating factor (GM–CSF), and tumor necrosis factor-alpha (TNF-α) [14, 15]. In other means, NKT can stimulate Th1 and Th2 responses. There is some evidence for a protective role of NKT cell in MS and other autoimmune disease, however their possible role in NMOSD is unclear [16].

B lymphocytes are involved in regulating the severity of autoimmune diseases such as MS and NMOSD. B lymphocytes can be classified into subgroups 1 and 2. Conventional B cells (B2) activate and polarize T lymphocytes to Th1 and Th2 cells, respectively [17]. B-1 cells mediate various functions such as antigen presentation, cytokine release and auto antibody production [18]. There are certain functional differences between B1 and B2. B1 cells are derived from fetal liver and to a lesser extent from the bone marrow of adults, whilst B2 cells are derived mainly from the bone marrow [19, 20]. B1 cells have two principal subsets, B-1a expressing CD5 as a pan T cell marker and B-1b, which does not express CD5 marker. [21]. IL-10 is produced by different subsets of innate and adoptive immune cells including T CD4^+^, T CD8^+^ cells, dendritic cells, macrophages and B cells, which can also impair T cell proliferation [22–24]. As an anti-inflammatory cytokine, IL-10 regulate inflammatory immune responses via inhibition MHC and co-stimulatory molecule expression, as well as pro-inflammatory cytokines production such as IL-1, IL-6, IL-12, IL-18, and TNF-a [23, 25, 26]. There is some evidence of the stimulatory effects of IL-10 on the immune system. An increase in IL-10 levels in EAE animal model leads to Th2 and T regulatory (Treg) differentiation, which mediates inflammation in EAE animal model [27–29]. However, IL-10 exerts its neuro-protecting effect by preventing glutamate-induced neuronal apoptosis through restoring suppressed anti-apoptotic elements, namely Bcl-2 and Bcl-xl, and reducing caspase-3 expression [30]. Despite clinical similarities between MS and NMOSD, there are critical differences concerning the pattern of pathogenesis and therapeutic protocols that are used for their treatments [34]. Due to the immunological basis of the two diseases, a great deal of research has been carried out on the distinctive components of the immune system [31]. The aim of our study was to investigate the peripheral blood frequency of the CD3^−^CD56^+^CD16^+^NK, CD3^+^CD56^+^ NKT and CD5^+^CD19^+^ B cells and serum IL-10 in IFN-treated and untreated RRMS, NMOSD, and healthy individuals.

## Methods

### Blood samples

The study groups comprised: 15 RRMS (under treatment with interferon beta-1a for at least 1 year), 15 RRMS (early onset RRMS patients who were diagnosed with MS for less than 3 years, who were not receiving any immunomodulatory or immunosuppressive drugs), 15 NMOSD (on immunosuppressive treatment), and 30 HC subjects. The MS and NMOSD patients were diagnosed according to the McDonald 2010 and Wingerchuck criteria, respectively [32]. Moreover, all the individuals with NMOSD were NMO-IgG seropositive. The healthy subjects were matched in terms of age and sex. The control subjects had no history of autoimmune, inflammatory or malignant diseases. The study was approved by the Ethical Committee of the Iran University of Medical Science and informed consent was obtained from all participants. 5 ml peripheral blood was collected from all subjects. Samples were transferred to the laboratory and fresh whole blood was utilized for flow cytometry assay. Serum was separated and stored at − 20 °C for subsequent measurement using ELISA. The serum level of IL-10 was assessed by using IL-10 high sensitivity human ELISA kit (Abcam, usable range: 1.56–50 pg/ml; and a sensitivity of 1.3 pg/ml). All methods in this study were conducted based on the ethical standards of the local ethics committee of Iran University of Medical Sciences and also based on the 1964 Helsinki declaration, its recent amendments or comparable ethical standards.

### Sample staining and flow data analysis

The following flurochrome monoclonal antibodies were purchased from BD Bioscience Company: PerCP (Peridinin Chlorophyll Protein Complex) mouse anti-human CD3 clone SP34-2, FITC (Fluorescein isothiocyanate) mouse anti-human CD16 clone 3G8 and PE (phycoerythrin) mouse anti-human NCAM-1 (CD56) clone R19-760, PE mouse CD5 clone UCHT2 (RUO) and FITC mouse anti-human CD19 clone HIB19 (RUO). Isotype controls were also utilized (all the antibodies were purchased from BD Bioscience). FACSCalibur Flowcytometer (Becton Dickinson Immunocytometry Systems) was used in three- and two-color staining to quantitate NK, NKT and B cells, respectively. After staining, tubes were incubated for 30 min at 4 °C in dark, followed by the addition of 1 ml lysing buffer. The tubes were incubated for 15 min in dark and samples were prepared for survey by flow cytometry. After gating on the lymphocyte population, 100,000 cells were counted on each gate. All flow data were analyzed using Flowjo 10 version.

### Gating strategy

**Fig. 1:**
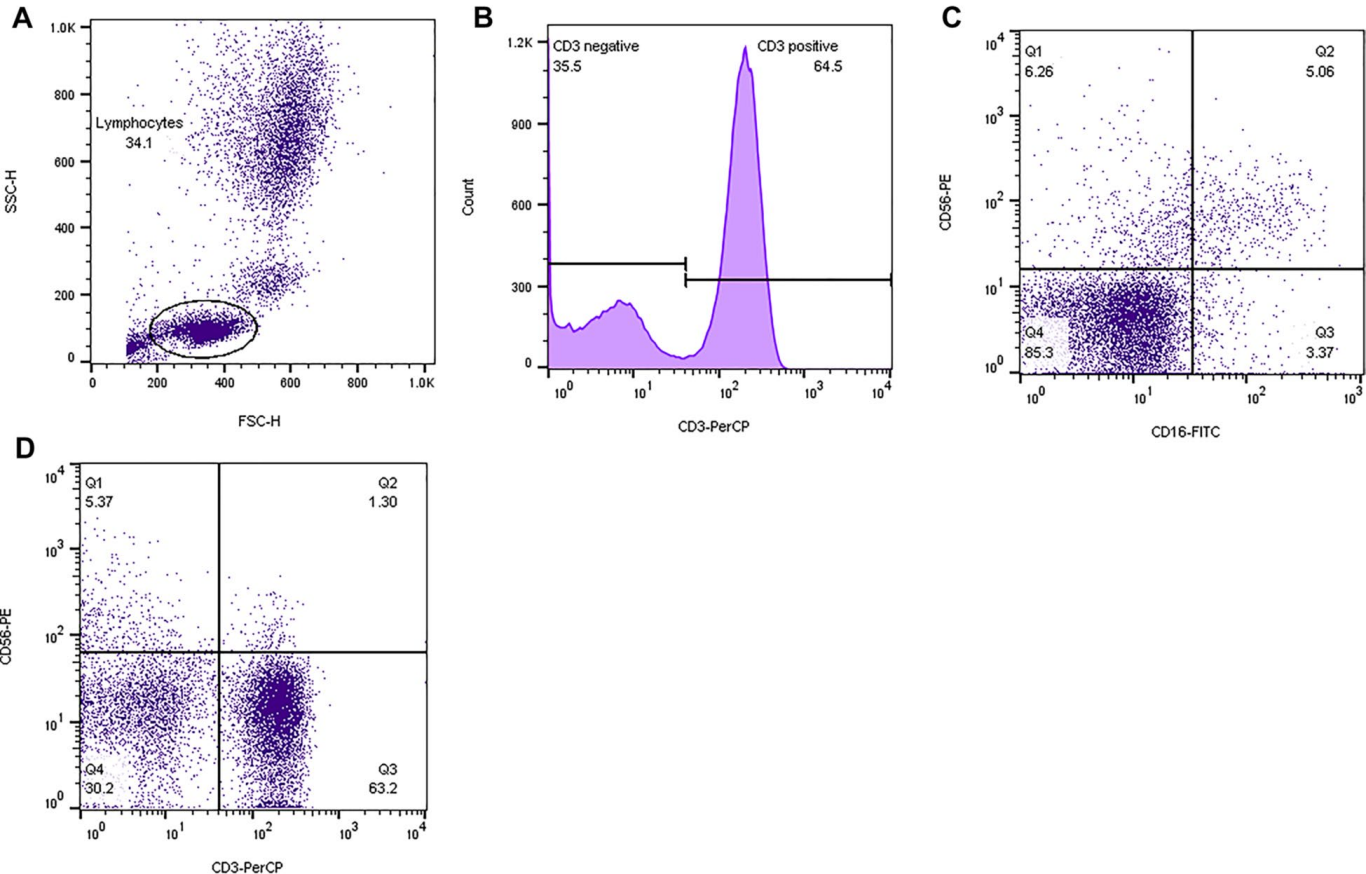
Flow cytometry analysis of NK cells. **A** Whole blood pattern, Lymphocyte gating. **B** We gated CD3 negative population for NK cells analysis. **C** As shown, there are different populations: CD3^−^ CD56^+^ (Q1), CD3^−^ CD16^+^ CD56^+^ (Q2), and CD3^−^ CD16^+^(Q3) NK cells. **D** After lymphocyte gate, we drew a dot plot of CD3^+^ CD56^+^ cells, which represent the NKT cells. This is a data of HC subject

To analyze the NK cells, we first gated the lymphocyte in the whole blood population using forward and side scatters plot (Fig. 1A), then the CD3 negative population was gated to analysis different population of NK cells (Fig. 1B). Q2 with CD3^−^CD16^+^CD56^+^ phenotype is known as NK cells. A sum of Q3 and Q2 is equal to the percentage of CD3^−^CD16^+^ cells. In addition, Q1 plus Q2 cells are representative of CD3^−^CD56^+^ cells. (Fig.1C). In order to analyze NKT cell, after gating lymphocyte population in forward and side scatters plot, a quadrant was drew in CD3 and CD56 dot plots and dual population of CD3^+^CD56^+^ (Q2) was considered as CD3^+^CD56^+^ NKT cells (Fig. 1D).

**Fig. 2:**
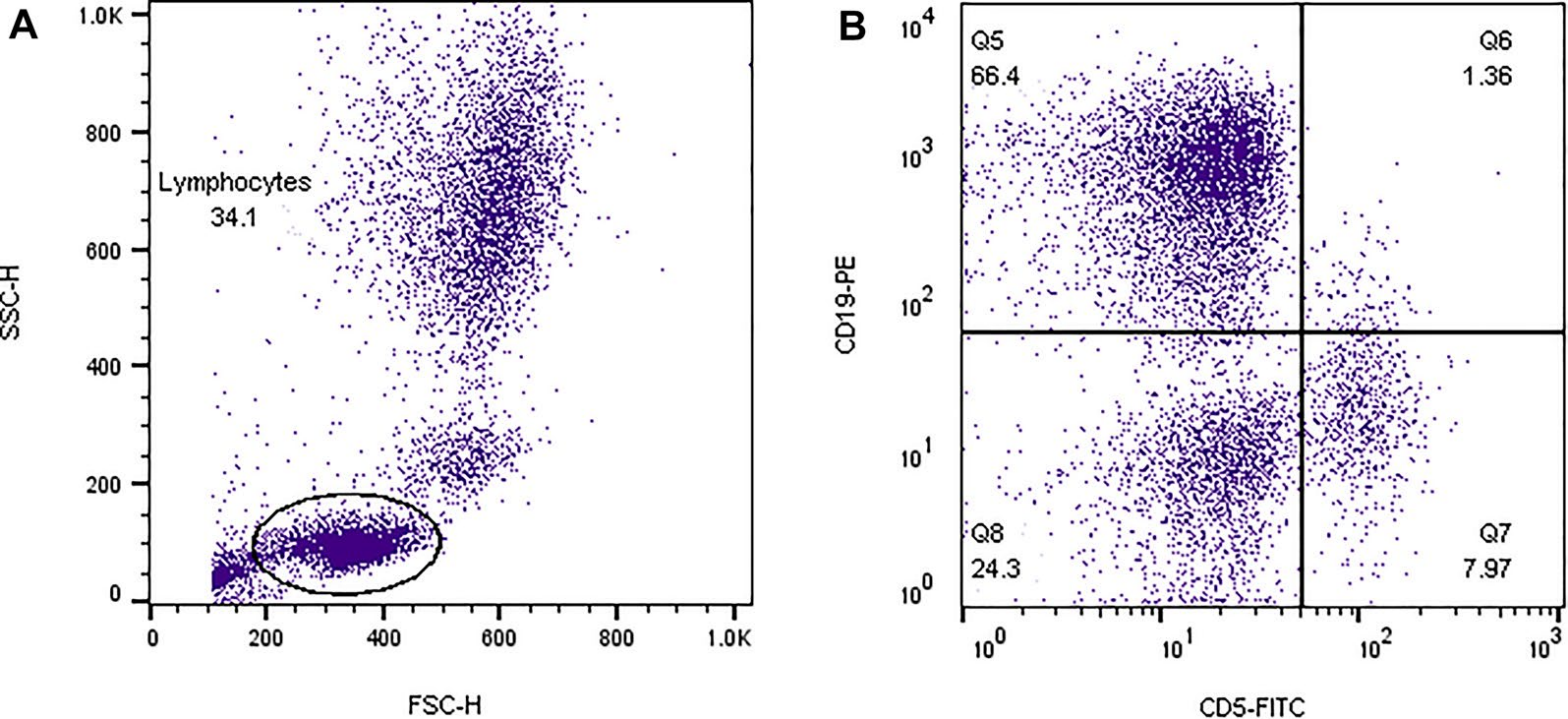
Flow cytometry analysis of B1 cells. **A** Whole blood pattern, Lymphocyte gating. **B** A quadrant was drew and double population of CD5 CD19 as Q6 are shown This data represents HC

For B cells analysis, following lymphocyte gating (Fig. 2A), a dot plot of CD5 and CD19 parameters was drew and dual CD19^+^CD5^+^ population (Q2) was considered as CD5^+^CD19^+^ B1 cells (Fig. 2B).

### Statistical analysis

The normality in the distribution of data, was assessed by Shapiro–Wilk test. A one-way ANOVA test was then employed and a P value ≤ 0.05 was considered significant. Data are showed as mean ± SD. All analyses were conducted by Graphpad Prism.

## Results

### Comparison of NK and NKT cells among the groups

**Fig. 3:**
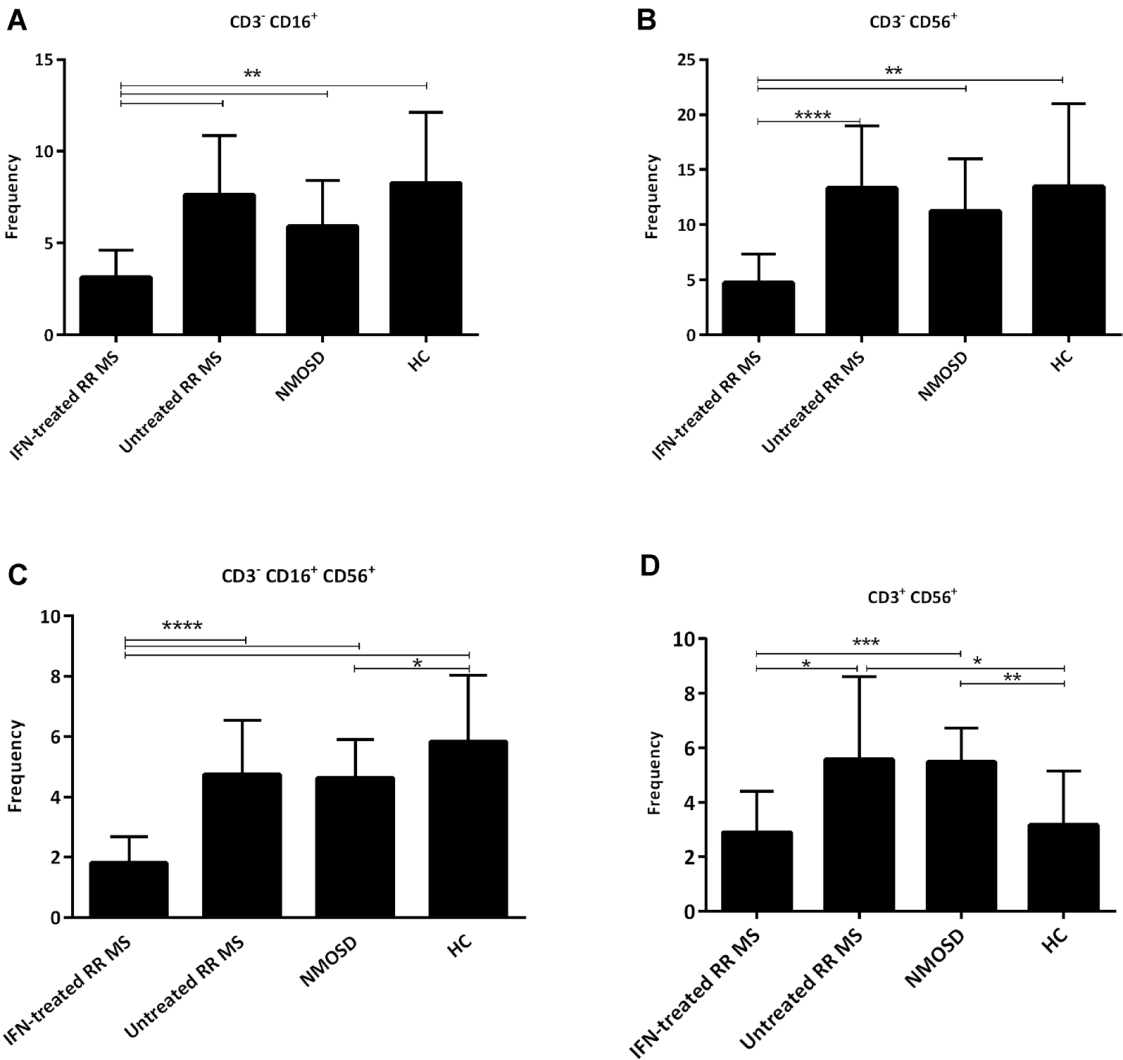
Comparison the mean percentage of **A** CD16, **B** CD56, and **C** CD56^+^CD16^+^, NK Cells among the groups $\pm$ SD

CD3-CD16^+^CD56^+^, CD3^−^CD16^+^, CD3^−^CD56^+^, CD3^+^CD56^+^ expression was assessed in the peripheral blood of IFN-treated RRMS [15], untreated RRMS [15], NMOSD [15] and healthy [30] subjects using a FACS Calibur flow cytometer, as shown in Table 1. Table 2 shows age and sex distribution among the groups. No significant differences in patients Expanded Disability Status Scale (EDSS) score were observed (Table 2). Based on Tables 1 as well as Fig. 3C, there was a lower percentage of CD3^−^CD56^+^CD16^+^ cells in IFN-treated RRMS (1.81 ± 0.87) compared to untreated RRMS (4.74 ± 1.80), NMOSD (4.64 ± 1.26) and HC (5.83 ± 2.19) groups (p < 0.0001). CD3^−^CD56^+^CD16^+^ percentage was significantly lower in NMOSD than HC groups (p < 0.047). Similarly, CD3^−^CD16^+^ and CD3^−^CD56^+^ proportion was in the lowest level in IFN-treated RRMS (3.13 ± 1.49, 4.73 ± 2.63; respectively) compared to other groups (p < 0.0001 and p < 0.0007, respectively; Fig. 3A and B). As shown in Table 1, the percentage of CD3^+^CD56^+^ cells were significantly higher in number in the untreated RRMS (5.56 ± 3.04) and NMOSD (5.47 ± 1.24) compared to the IFN-treated RRMS (2.89 ± 1.51) groups (p < 0.05 and p < 0.001; respectively) (Table 1, Fig. 3D). There was a significant difference in CD3^+^CD56^+^ between untreated RRMS and HC (p < 0.05) as well as between NMSOD and HC (3.16 ± 1.98) (p < 0.01) (Table 1, Fig. 3D).

**Table 1:** CD3^−^ CD16^+^, CD3^−^ CD56^+^, CD3^−^ CD16^+^ CD56^+^ NK cells, CD3^+^ CD56^+^ NKT cells, and CD5^+^ CD19^+^ B cells percentage, IL-10 serum levels in IFN-treated RRMS, IFN-treated MS, NMOSD and HC groups

Variables	IFN-treated RR MS [15]	Untreated RR MS [15]	NMOSD [15]	HC [30]	P value
CD3^−^CD16^+^ CD56^+^	1.81 $\pm$ 0.87	4.74 $\pm$ 1.80	4.64 $\pm$ 1.26	5.83 $\pm$ 2.19	< 0.0001
CD3^−^CD16^+^	3.13 $\pm$ 1.49	7.62 $\pm$ 3.23	5.91 $\pm$ 2.50	8.28 $\pm$ 3.85	< 0.0001
CD3^−^CD56^+^	4.73 $\pm$ 2.63	13.31 $\pm$ 5.67	11.23 $\pm$ 4.76	13.49 $\pm$ 7.51	< 0.0007
CD3^+^CD56^+^	2.89 $\pm$ 1.51	5.56 $\pm$ 3.04	5.47 $\pm$ 1.24	3.16 $\pm$ 1.98	< 0.002
CD5^+^CD19^+^ B cell	0.81 $\pm$ 0.17	1.32 $\pm$ 0.67	0.30 $\pm$ 0.20	0.5 $\pm$ 0.22	< 0.0001
Serum level of IL-10 (pg/ml)	8.06 $\pm$ 5.39	5.07 $\pm$ 1.44	5.33 $\pm$ 2.56	8.38 $\pm$ 2.84	< 0.003
Total number of attack in first two year	1.89(1–4)	2.5(1–5)	2.01(1–4)	–	N.S
Disease duration	5.6(0.4–13)	2.6(0.1–10)	6.6(1–17)	–	0.04
Age of disease onset	29.8(16–56)	25.6(12–44)	30.8(19–42)	–	N.S

Values indicate mean $\pm$ SD. The values of three last parameters show mean (min–max). The flow cytometric result are shown as percentage

*IFN-treated* interferon-β treated, *RRMs* relapsing remitting multiple sclerosis, *NMOSD* neuromyelitis optica spectrum disorder, *HC* healthy control, *NK* natural killer, *IL-10* interleukin 10

**Table 2:** Clinic-demographic data. EDSS and age values indicate mean $\pm$ SD

	IFN-treated RR MS	Untreated RR MS	NMOSD	HC
Gender				
(F)	12	9	12	20
(M)	3	6	3	10
EDSS	1.4 $\pm$ 1.69	2.1 $\pm$ 1.72	1.5 $\pm$ 1.64	0
Individual	15	15	15	15
Mean age	34 $\pm$ 9.93	33 $\pm$ 7.52	38 $\pm$ 8.23	30 $\pm$ 8.85

*IFN-treated* interferon-β treated, *RRMs* relapsing remitting multiple sclerosis, *NMOSD* neuromyelitis optica spectrum disorder, *HC* healthy control, *F* female, *M* male, *EDSS* expanded disability status scale

### Comparison of CD5^+^CD19^+^ B cell and IL-10 among the groups

**Fig. 4:**
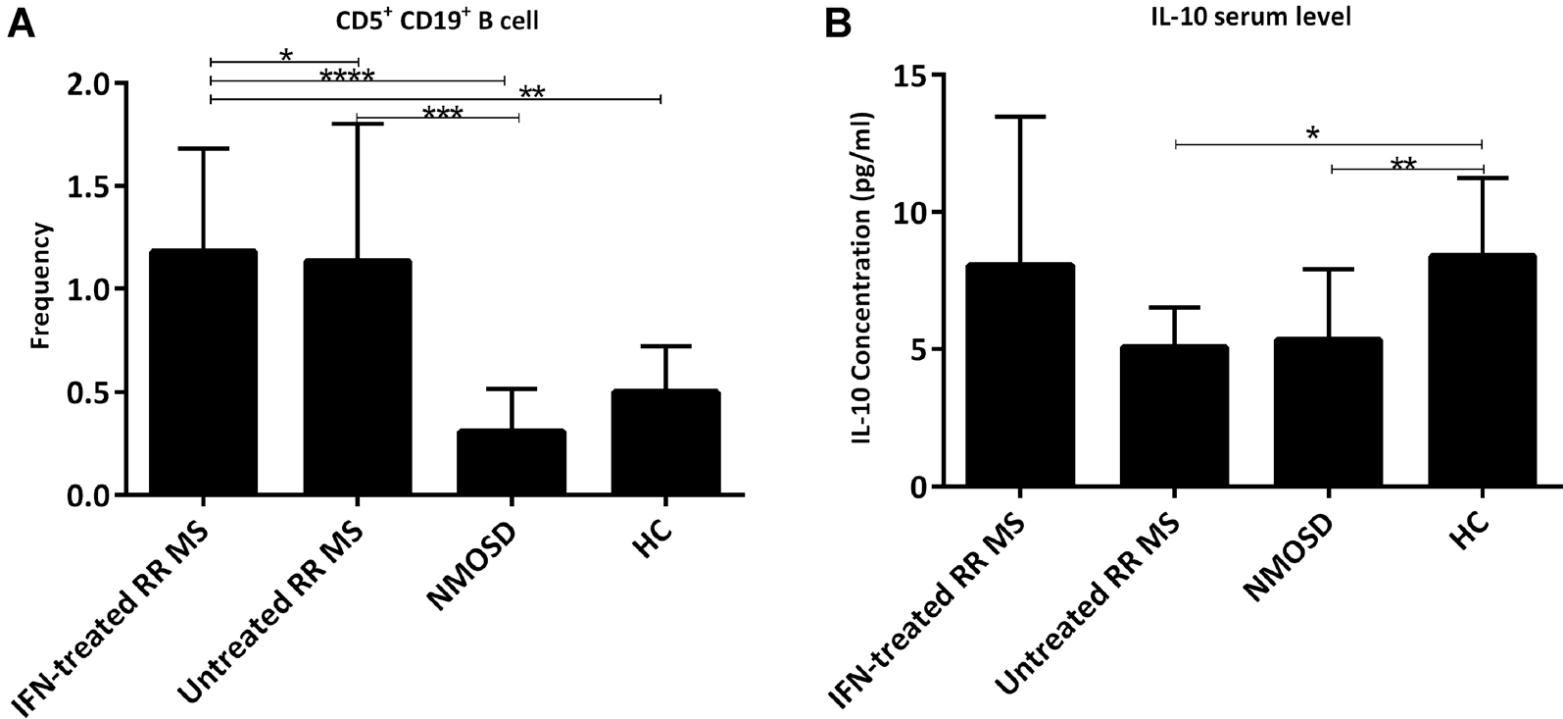
Comparison the mean percentage of **A** CD5^+^CD19^+^ cell, **B** IL-10 serum level among the groups $\pm$ SD

The frequency of CD19^+^CD5^+^ B cells was significantly different among the groups (Table1, p < 0.05). The lowest percentage of CD19^+^CD5^+^ B cells was observed in the patients with NMOSD (0.30 ± 0.20). Untreated RRMS (1.32 ± 0.67) and IFN-treated RRMS (0.81 ± 0.17) group showed a higher frequency in CD19^+^CD5^+^ B cells compared to the HC (0.5 ± 0.22) group (Fig. 4A). IL-10 serum levels were also significantly different among the groups (Table 1, Fig. 4B; p < 0.003), the lowest IL-10 levels was observed in untreated RRMS (5.07 ± 1.44 pg/ml) individuals, while IL-10 levels of IFN-treated RRMS and HC were at higher frequencies (8.06 ± 5.39 pg/ml and 8.38 ± 2.84 pg/ml, respectively). A significant difference was observed between HC and NMOSD (5.33 ± 2.56) (p < 0.01) as well as untrated RRMS (p < 0.05). No significant correlation was observed betweeen measured paramteres.

## Discussion

NK cells play a vital role in immune defence against micro-organisms and the regulation of immunological responses against tumors. NK cells have been noted for their involvement in immunological aspects of multiple sclerosis. There are several reports about the effect of NK and NKT cells in neuroimmunological aspect of multiple sclerosis disorder. The controversial reports of NK cells motivated us to study them in two autoimmune diseases in different groups as well as healthy subjects [33, 34]. In the present study, the minimum and maximum percentage of CD3^−^CD16^+^, CD3^−^CD56^+^, and CD3^−^CD16^+^CD56^+^ was observed in IFN-treated RRMS and HC, respectively. The present results are similar to previous reports that have reported the importance of interferon beta therapy in MS patients to reduce CD3^−^CD56^+^ NK cell in the peripheral blood [35, 36]. A signifiant decrease in CD3^−^CD16^+^CD56^+^ NK cell in NMOSD compared to HC group (p < 0.05, Table 1, Fig. 3C) was observed in our study. Nevertheles, Tahrali et al. in the investigation of surface markers and intracellular cytokine of NK cells stated that the level of inflammatory responses of NK cell is significantly higher in RRMS treated compared to untreated RRMS patients. They included IFN and glatiramer acetate (GA) in treated RRMS patients, whereas we studied purticularly IFN-treated RRMS patients [37]. A previous study reported that GA leads to rise in NK cell activities by increasing cytotoxicity receptors such as NKp30, NKp44, NKp46 and NKG2D. The discrepency identified above may be largly bacause of the treatment used [38]. Ding et al. recently published that the frequency of NK cell in remission and acute phase of NMOSD is significantly lower than acute phase MS patients [39]. Although no significant differences were observed between NMOSD and untreated RRMS patients in the present study, which may be due to the different number of patients and not dividing the NMOSD group into subjects with acute disease or in remission. According to Mandal et al. NK cell proportion is higher in the onset of disease, when it is hard to evaluate these cells [40]. An animal study using the EAE model revealed the severity of disease could be ameliorated by NK cell depletion [6, 41, 42], which highlight the protective role of NK cells. These kinds of discerepencies emphasize on the great imortance of clarifing the distinct phenotype of NK cell in different phase of disease.

Moreover, our results showed no significant difference in CD3^−^CD16^+^CD56^+^ between untreated RRMS and HC groups, which is consistent with the study of Laroni et al.; in which they reported that the regulatory function of these cells is diminished [43].

The proportion of CD3^+^CD56^+^ cell in peripheral blood of untreated RRMS patients was higher than HC and IFN-treated RRMS (both p < 0.05). Interestingly, this population was more frequent in NMOSD compared to IFN-treated RRMS and HC (p < 0.001 and p < 0.01; respectively). This decline in NKT cell proportion in IFN-treated RRMS is confirmed by previous studies [44, 45]. However, O'Keeffe et al. reported NKT cell frequency was higher in RRMS than in HC [46]. CD1d-deficient EAE mice showed more severe symptom of disease, the protective role of NKT in EAE model is maybe due to inhibiting Th1 responses by Th2 cytokine profile [47]. There is no direct evidence about CD3^+^CD56^+^ cells in NMOSD.

B cells are generally involved in shaping specific immune responses such as plasma cell differentiation, auto antibody and cytokine production [48]. They act as antigen presenting cells to stimulate T cell responses. These mechanisms may contribute to MS disease pathogenesis [49]. EAE animal studies have verified the pathogenicities of B cells by means of their differentiating into plasma cells and auto antibody production [50]. CD5^+^ B-1 cells are long-living and self-renewing subsets of B cells with high capability in poly reactive auto-antibody production and apoptotic bodies clearance. Since autoantibodies are secreted by B-1 cells, they are regarded as potential contributors to several autoimmune diseases, such as MS, Sjogren’s syndrome, SLE and rheumatoid arthritis [51]. CD5 is one of the critical marker of B1 cells, which is involved in regulating several immune cells. We conducted an investigation on peripheral blood levels of CD19^+^CD5^+^ B1 cells in IFN-treated and untreated RRMS, NMOSD and HC groups for the first time and a significant difference between groups was demonstrated. Untreated RRMS subjects had the highest share of B1 cells (p < 0.0001). This was the first time that the frequency of CD19^+^CD5^+^ cells was investigated in NMOSD patients, and according to the results, it was significantly lower than for healthy subjects (Fig. 4A, Table 1). The lower levels of B1 cells were validated in a study by Rovituso et al., in which the peripheral blood levels of CD20^+^CD27^+^CD43^+^CD70^−^ B1 cells were lower in MS patients in contrast to HC. These results may shed further light on the important role of B-1 cells in MS and NMOSD [52]. There are some supporting evidence about the involvement of B-1 cells in MS, as Torring et al. reported the elevated frequency of B1 cells in the remiting phase of MS condition [53]. It is reported that the frequency of B1 cells are elevated in autoimmune disorders in human and mouse model and genetic depletion of this cell population in mouse models led to ameliorate autoimmunity [17, 54]. Due to the paucity of studies, drawing a precise conclusion about the disparity of B1 cell in neuro autoimmune diseases, particulary NMOSD needs furthur studies. However, it seems that B1 cells may contribute to the immunomodulatory status of the immune system, which favors better recovery from MS autoimmunity. Apparently, more inquiries would provide supplementary details on B1 cell functions. Pre-naive mature B cells, that express CD5 marker, play their role in humoral immunity via their differentiation into plasma cells and antibody production [55]. Mature B cells also are involved in T cell immunity formation by presenting antigen, supplying them stimulation and cytokines, and activating them to develop effector and memory T cells [56]. Moreover, B cells can affect immune responses by inhibiting TCD4^+^ cells formation and Treg induction to secrete IL-10 [57]. CD5 marker of B1 cells is contributor to IL-10 production through a non-selective calcium ion channel named transient receptor potential channel 1. Upon this Ca^2+^ dependent pathway, mitogen-activated protein kinase is activated, which leads to phosphorylation of extracellularly-regulated kinase-1 and -2 Erk1/2 and IL-10 procuction [58]. Moreover, IL-10 is highly engaged in inhibition of auto-reactive B1 cells via a negative feedback [54, 59]. Various subsets of leukocytes are invovled in IL-10 production including Th1, Treg, TCD8^+^, B cells, macrophages, dendritic cells, neutrophils and eosinophils [60]. IL-10 manage immunusupressive effects via blocking the process of immunce response foramation for instance B7/CD28 costimulatory pathway and DC maturation, which results in decreasing the MHC class II expression [61]. IL-10 has controversial effects on different autoimmune diseases (SLE vs. MS). B-1 cells can exacerbate several disorders such as SLE, due to the production of cytokines and natural autoantibodies as well as their antigen presenting capabilities [62]. To evaluate the balance between Th1 and Th2 responses, we selected IL-10 as a surrogate of Th2 immunity. Lower levels of serum IL-10 were observed in MS and NMOSD patients compared to HC (p < 0.05). Intrestingly, it was demonstrated that the serum levels of IL-10 were higher in IFN-treated RRMS compared to untreated RRMS patients (Fig. 4B). This possibly suggests the regulatory function of IL-10 in MS disease. Furthermore, there is a less severe inflammatory response in the remission phase of MS compared to the relapsing phase. Previous investigation has been elucidated that IL-10 serum level would decrease before relapse and increase in remission phase of MS [63]. In the other word, regarding to a prior study the IL-10 levels decline during relapse and elevate through remission, demonstrating the necessity of IL-10 during the remission phase [64]. This could confirm a shift from Th responses toward Th2 dominance after MS first line therapy, which was administered in naive MS patients, who did not receive any immunomodulatory drugs. Interestingly, Wei et al. reported clinically isolated syndrome patients with lower IL-10 level were more predicted to develop secondary relapse [65]. However, Kallaur et al. stated no significant difference in IL-10 levels between MS and HC groups [66]. It is suggested to consider more cytokines especially intracellular as well as regulatory and activatory markers in the future studies. To explain in detail, it is highly recommended to investigate some regulatory markers including FoxP3 in CD5^+^ B cells along as intracellular level of lL-10. Adding more markers would clarify their functions in more features. It is also applies to NK cells. Investigating the different subsets of NK and B1 cells, including more inflammatory and non-inflammatory cytokines, and their possible roles in neuro-autoimmune conditions would elucidate more detailed information about their possible correlation with disease severity.

## Conclusions

The lower proportion of CD3^−^CD56^+^CD16^+^ NK and CD3^+^CD56^+^ cells in the peripheral blood of IFN-treated patients with RRMS compared to other groups suggests the importance of immunomodulation in patients with RRMS disorder. Based on the differences in CD19^+^CD5^+^ B cells and serum IL-10 between patients and HC, supplementary assessments could be of value in clarifying their roles in autoimmunity. This approach could be productive in treatment prediction and development of novel therapies.

## Data Availability

All data generated or analyzed during this study are included in this published article.

## Abbreviations

MS: Multiple sclerosis
NMOSD: Neuromyelitis optica syndrome disease
EDSS: Expanded disability status scale
CNS: Central nervous system
NK: Natural killer cells
HC: Healthy controls
IL-10: Interleukin-10
Th: T Helper
Treg: T regulatory
FNγ: Interferon gamma
TNF- α: Tumor necrosis factor alpha
GM–CSF: Granulocyte–macrophage colony-stimulating factor
IL-13: Interleukine-13
ELISA: Enzyme-linked immunosorbent assay
QP4: Aquaporin-4
